# Endocannabinoids are Involved in Male Vertebrate Reproduction: Regulatory Mechanisms at Central and Gonadal Level

**DOI:** 10.3389/fendo.2014.00054

**Published:** 2014-04-15

**Authors:** Patrizia Bovolin, Erika Cottone, Valentina Pomatto, Silvia Fasano, Riccardo Pierantoni, Gilda Cobellis, Rosaria Meccariello

**Affiliations:** ^1^Department of Life Sciences and Systems Biology, University of Turin, Turin, Italy; ^2^Neuroscience Institute of Turin, University of Turin, Turin, Italy; ^3^Dipartimento di Medicina Sperimentale, Seconda Università di Napoli, Naples, Italy; ^4^Dipartimento di Scienze Motorie e del Benessere, Università di Napoli Parthenope, Naples, Italy

**Keywords:** GnRH, hypothalamus, pituitary, spermatogenesis, chromatin remodeling, male fertility

## Abstract

Endocannabinoids (eCBs) are natural lipids regulating a large array of physiological functions and behaviors in vertebrates. The eCB system is highly conserved in evolution and comprises several specific receptors (type-1 and type-2 cannabinoid receptors), their endogenous ligands (e.g., anandamide and 2-arachidonoylglycerol), and a number of biosynthetic and degradative enzymes. In the last few years, eCBs have been described as critical signals in the control of male and female reproduction at multiple levels: centrally, by targeting hypothalamic gonadotropin-releasing-hormone-secreting neurons and pituitary, and locally, with direct effects on the gonads. These functions are supported by the extensive localization of cannabinoid receptors and eCB metabolic enzymes at different levels of the hypothalamic–pituitary–gonadal axis in mammals, as well as bonyfish and amphibians. *In vivo* and *in vitro* studies indicate that eCBs centrally regulate gonadal functions by modulating the gonadotropin-releasing hormone–gonadotropin–steroid network through direct and indirect mechanisms. Several proofs of local eCB regulation have been found in the testis and male genital tracts, since eCBs control Sertoli and Leydig cells activity, germ cell progression, as well as the acquisition of sperm functions. A comparative approach usually is a key step in the study of physiological events leading to the building of a general model. Thus, in this review, we summarize the action of eCBs at different levels of the male reproductive axis, with special emphasis, where appropriate, on data from non-mammalian vertebrates.

## Introduction

Since the discovery of Δ^9^-tetrahydrocannabinol (THC) as the main psychoactive ingredient in marijuana, the subsequent cloning of cannabinoid receptors and the identification of their endogenous ligands [i.e., endocannabinoids (eCBs)], our understanding of the functions of the eCB system (ECS) has evolved considerably. It has become evident that most components of the mammalian ECS are highly conserved in evolution, pointing to a fundamental modulatory role in basic cellular and organismic functions (1, 2). Accordingly, the ECS is widely expressed in vertebrates, central and peripheral organs, and regulates a large array of physiological functions and behaviors.

The basic eCB signaling system consists of (1) at least two G-protein-coupled receptors, known as the cannabinoid type-1 and type-2 receptors (CB1 and CB2); (2) the endogenous ligands, of which anandamide (AEA) and 2-arachidonoylglycerol (2-AG) are the best characterized; and (3) synthetic and degradative enzymes and transporters that regulate eCB levels and action at receptors. CB1 receptors are abundant in the whole vertebrate central nervous system (CNS) and some peripheral tissues (3–5), whereas CB2 receptors are mostly expressed in peripheral tissues and immune cells, but they have recently been found also in the CNS (6–8). Research in mammals has provided evidence that eCBs can also bind to and activate type-1 transient receptor potential vanilloid (TRPV1) channels (9).

An enormous amount of information on the general properties of the ECS has accumulated over the last two decades [for general reviews on the ECS, see Ref. (10–14)]. In the past years, growing evidence has been accumulating to show the central role of the ECS in controlling vertebrate reproductive functions at both central and gonadal level (15). This review will summarize the action of eCBs at different levels of the reproductive axis, including data from non-mammalian vertebrates.

## Effects of eCBs on Hypothalamic–Pituitary Control of Reproduction

Reproductive functions are under neuroendocrine control and require a tight crosstalk between the hypothalamus, pituitary, and gonads. Gonadotropin-releasing-hormone (GnRH) is a key molecule in reproductive behavior and physiology. This neuropeptide is synthesized by hypothalamic neurons mostly located, in mammals, in the preoptic area and in the arcuate nucleus. GnRH axons project to the median eminence, where pulsatile release of GnRH into the hypophysial portal circulation drives the synthesis and secretion of follicle-stimulating hormone (FSH) and luteinizing hormone (LH) from anterior pituitary gonadotropic cells. Circulating FSH and LH, in turn, stimulate gametogenesis and the synthesis and secretion of the gonadal steroid hormones, androgens, estrogens, and progesterone. Under various physiological and pathological conditions, hormonal and metabolic signals regulate GnRH neurons both directly or through upstream neuronal circuitries to influence the pattern of GnRH secretion. The emerging picture from studies in different vertebrate models is that eCBs can modulate both GnRH and gonadotropic cell function, in other words that eCBs can influence the regulation of reproduction at both hypothalamic and pituitary levels (16, 17).

There is general agreement on the inhibitory effect exerted by cannabinoids and eCBs on GnRH release. Early studies in rats demonstrated that the ECS influence gonadal androgens via effects on the hypothalamus and the anterior pituitary. THC, as well as eCBs, lowers not only circulating testosterone levels but also the levels of LH and FSH (18). Most of this negative effect appears to be exerted by inhibition of GnRH secretion into median eminence blood portal vessels (19, 20). Serum LH decreases in response to AEA administration in wild-type mice, whereas CB1 knockout mice (*Cb1*^−/−^) are unresponsive to the treatment (21) and show low levels of GnRH and FSH-beta mRNA at hypothalamic and pituitary levels (22), demonstrating the pivotal role exerted by CB1 in the regulation of GnRH and godanotropins synthesis and/or release.

The above effects require CB1 expression in ventro-medial telencephalic and hypothalamic regions. Early localization studies in rodents detected a low abundance of CB1-immunoreactive axons (23) and a low expression level of *CB1* mRNA (24–26) in the rodent hypothalamus. However, more recent immunocytochemical studies (27) revealed a dense CB1-immunoreactive fiber network in the mouse hypothalamus. These data are consistent with studies in teleosts and amphibians, showing the expression of CB1-immunoreactive fibers and cell bodies in several hypothalamic regions of adult teleosts (*Carassius auratus* and *Pelvicachromis pulcher*) and anuran amphibians (*Xenopus laevis* and *Rana esculenta*) (4, 5, 28, 29), as well as in zebrafish and in embryos of *X. laevis* (30, 31). The expression of *CB1* appears to be regulated in the diencephalon during the annual sexual cycle in anuran amphibians (32). Interestingly, *CB1* fluctuations show an opposite trend compared to *GnRH-I* mRNA variations, suggesting that maximal GnRH release corresponds to minimal CB1 levels in the diencephalon. Both *GnRH-I* and *GnRH-II* expressions are inhibited in the frog diencephlaon by AEA administration, indicating that both molecular forms might be involved in the regulation of gonatropin discharge (33). Only few data so far indicate that CB2 and TRPV1 receptors might have a role in GnRH cell regulation. Profiling neurotransmitter receptor expression in mouse GnRH-secreting neurons revealed CB2 expression in diestrous adult females (34), and CB1/TRPV1 co-localization has been reported in mouse hypothalamic paraventricular nucleus (35).

An important question is whether eCBs exert their effect directly on GnRH neurons, or on neighboring cells that control GnRH release. Gammon et al. (36) demonstrated that immortalized GnRH neurons (GT1 cells) are both a source and target of eCBs; they produce and secrete 2-AG and AEA, are able to take up and degrade eCBs, and possess CB1 and CB2, whose activation leads to the inhibition of pulsatile GnRH release. Nevertheless, such observations have not been confirmed *in vivo* in mammals, although GnRH-secreting neurons are close to cannabinergic fibers in male mice (37) and few hypothalamic GnRH neurons seem to express CB1 receptors (36). Close proximity between CB1-expressing fibers and GnRH cells has been well documented in non-mammalian vertebrates. In *P. pulcher*, *C. auratus*, *Solea solea*, and *Danio rerio*, CB1-containing cell bodies and terminals codistribute with GnRHIII (also called *salmon* GnRH) cell bodies and fibers (38–40). Similarly, codistribution of CB1- and GnRH-I-immunoreactivity has been found in corresponding brain regions of *X. laevis* and *R. esculenta* (39, 41). Noteworthy, a subset of frog GnRH-I-immunoreactive neurons in the septum and preoptic area are also CB1 immunopositive (28), suggesting the existence of a CB1-mediated autocrine mechanism in the control of GnRH secretion, in addition to presynaptic mechanisms. Ultrastructural studies in mammals indicate that CB1-immunoreactive terminals establish symmetric as well as asymmetric synapses on GnRH neurons, suggesting that retrograde eCB signaling might influence GABAergic and glutamatergic synaptic transmission, respectively (27). It should be noted that most recent studies examining the effects of endogenous GABA release on GnRH neurons indicate that the predominant action is that of excitation (42). In line with this, Farkas et al. (37) provided electrophysiological and morphological evidence that retrograde eCB signaling reduces GABAergic excitatory drive onto GnRH neurons via activation of presynaptic CB1 receptors, and that the reduced GABA_A_ receptor signaling in turn inhibits GnRH neuron firing activity. Besides the major afferent regulation exerted on GnRH neurons by GABAergic and glutamatergic inputs, available neuroanatomical literature describes afferent inputs by peptidergic and monoaminergic neuronal systems (43). However, whether the ECS interacts also with these systems has not been determined yet.

Besides the effect on GnRH cells, eCBs could also modulate the activity of other hypothalamic cell types involved in reproduction. Cells containing aromatase, the enzyme that catalyzes the transformation of androgen into estrogens, are localized in the hypothalamus and are deeply involved in sexual differentiation of the brain and activation of male sexual behavior. Aromatase and CB1 are expressed in close contiguity in the goldfish preoptic area and periventricular gray of hypothalamic inferior lobes (16), suggesting a possible CB1-mediated regulation of aromatase activity, at least in bony fish.

Several lines of evidence indicate that eCBs may control adenohypophyseal hormone secretion also acting directly at pituitary level. Both AEA and 2-AG have been detected in the anterior pituitary, suggesting local synthesis (44). In addition, CB1 has been localized in the anterior pituitary within the gonadotroph and lactotroph cells in adult male rats (45, 46), in humans (47), and in *X. laevis* (48). CB1 expression in pituitary depends on steroids, since it is reduced in both orchidectomized male and estradiol-replaced OVX female rats (46). Recently, the presence of ECS has been demonstrated in mammalian pars tuberalis (49). This finding might be functionally significant also for GnRH release, since this pituitary region is a key station for the anterograde signaling toward the pars distalis.

## Effects of eCBs at Gonadal Level

Beside the role exerted at hypothalamic level to control reproductive activity in both sexes, the discovery of eCBs in gonads and reproductive fluids – from seminal plasma in males to oviductal fluid and milk in females – (50–52) pointed out the importance of eCB signaling in the gonads. Gonads have the ability to synthesize eCBs which in turn exert differential effects activating both different types of receptors or tissue-/cell-specific receptor subtypes, the latter obtained by both alternative splicing or transcription sites (53–55). The content of eCBs is regulated by biosynthetic/hydrolyzing enzyme balance, and the appropriate “eCBs tone” *in loco*, is critical for spermatogenesis progression in male and follicle maturation in female, for sperm quality and the acquisition of sperm functions related to fertilization (motility and capacitation), for fertilization, early-embryo migration, implantation and placentation, for parturition onset and labor as well (15, 17, 56–63). Focusing on males, evidence of eCB direct action into the testis has been provided in most vertebrates [fish (8, 64), frogs (32, 57, 65–68), mammals (21, 69–74)), whereas an ECS has also been described in spermatozoa (SPZ) collected from sea urchin (75), amphibians (65), rodents (76–79), bull (80), boar (81), and human (82–85). A specific and significant association between the use of marijuana and the occurrence of non-seminomatous and mixed testicular germ cell tumors (TGCT) has been recently reported in humans (86–88); although a deep characterization of ECS has never been provided in TGCT patients yet, these data may suggest that the recreational and therapeutic use of cannabinoids may represent a risk factor for TGCT. In general, a relationship between the expression of cannabinoid receptors and the outcome of sex-steroid-dependent cancer has been documented, thus the imbalance in the ECS and its interaction with sex-steroid hormone homeostasis may promote cancer development, proliferation, and migration [for recent review, see Ref. (89)]. Defects in eCB signaling or eCB tone have recently been reported in rat treated with HU210 – a synthetic analog of THC – (90) as well as in clinical cases of male infertility in humans (85, 91). Consistently, genetic inactivation of the AEA-hydrolyzing enzyme, *Faah* (Fatty acid amide hydrolase) results in increased levels of AEA in the male reproductive system that negatively affect sperm motility and impair sperm fertilizing ability (92), whereas defects in the acquisition of sperm motility during the epididymal transit have been reported in *Cb1*^−/−^ mice (76, 77). Thus, ECS is nowadays considered a potential therapeutic target in male infertility. ECS is widely expressed in testis in both germ and somatic cells, and a map of ECS localization in several species is provided in Table 1. The first intratesticular targets of eCBs to be identified were the Leydig cells (21, 93), consistent with the low basal testosterone production observed in both *Cb1*^−/−^ mice and AEA-treated controls, providing evidence of mechanisms other than the AEA/THC-dependent downregulation exerted at hypothalamic/pituitary levels. The direct effect of Bhang (cannabis) on 3β-HSD, a well-known marker of Leydig cell activity, also confirmed this issue (79). The involvement of CB1 signaling in the control of Leydig cell activity is not restricted to steroid (both testosterone/estradiol) production (21, 22, 93), but also extends to Leydig cells ontogenesis. In fact, as reported by Cacciola et al. (72), CB1 expression in differentiating adult Leydig cells negatively correlates with cell division and the characterization of *Cb1*^−/−^ mice phenotype revealed a 30% decrease in Leydig cells number (72), as well as low circulating estradiol level (22) [for recent review, see Ref. (94)].

**Table 1:** **Localization of ECS components [both mRNA and protein (Prot)] in testicular somatic and germ cells**.

Cell type	CB1	CB2	TRPV1	FAAH	NAPE-PLD	MAGL	DAGLα/β	Species	Reference
Leydig cells	mRNA	Prot		Prot	mRNA			*R. esculenta*	(68, 69, 72, 79)
	Prot							*M. musculus*	
	Prot							*R. norvegicus*	
Sertoli cells	mRNA	mRNA/Prot	mRNA	mRNA/Prot	mRNA			*R. esculenta*	(68, 70–73)
	Prot							*M. musculus*	
								*R. norvegicus*	
ISPG	Prot	mRNA/Prot	mRNA	mRNA	mRNA	mRNA	mRNA	*R. esculenta*	(65, 69, 73)
	mRNA/Prot							*M. musculus*	
IISPG	mRNA/Prot	mRNA/Prot	mRNA	mRNA	mRNA	mRNA	mRNA	*R. esculenta*	(65, 68, 69, 73)
	mRNA/Prot				mRNA			*M. musculus*	
ISCP	Prot	mRNA/Prot	mRNA	Prot	mRNA	mRNA	mRNA	*R. esculenta*	(65, 68, 69, 73)
	mRNA/Prot			mRNA	mRNA			*M. musculus*	
IISPC	Prot	mRNA/Prot	mRNA	Prot	mRNA	mRNA	mRNA	*R. esculenta*	(65, 73)
	mRNA			mRNA				*M. musculus*	
SPT	mRNA/Prot	mRNA/Prot	mRNA	Prot	mRNA	mRNA	mRNA	*R. esculenta*	(65, 68, 69, 72, 73)
	mRNA			mRNA				*M. musculus*	
	Prot							*R. norvegicus*	
SPZ	mRNA/Prot	mRNA/Prot	mRNA/Prot	Prot	mRNA/Prot	mRNA/Prot	mRNA/Prot	*R. esculenta*	(65, 68, 72, 78, 81, 83, 84)
	mRNA/Prot Prot	Prot	Prot	mRNA/Prot	Prot			*M. musculus*	
	Prot	mRNA/Prot	Prot	Prot				*R. norvegicus*	
	mRNA/Prot	Prot		Prot				*S. scrofa*	
	mRNA/Prot			Prot				*B. taurus*	
								*H. sapiens*	

In the germinal compartment, AEA reduces the spermatogenic output inducing the apoptosis of Sertoli cells (70) in a mechanism reversed by FSH-dependent activation of aromatase and by estradiol-dependent upregulation of *Faah* (71). Recent studies carried out by Grimaldi et al. (95) demonstrated that in mature Sertoli cells *Faah* gene is a direct target of estradiol whose promoter contains two proximal estrogen-responsive element (ERE) sequences named ERE2/3. *In vivo*, a mechanism involving the binding of ERβ to ERE 2/3 and the epigenetic modifications of *Faah* gene proximal promoter (demethylation of both DNA at CpG site and histone H3 at lysine 9) has been demonstrated (95); consistently *FAAH* silencing abolished estrogen protection against AEA-dependent apoptosis (95). Thus, AEA content finely toned by its hydrolyzing enzyme FAAH is a fundamental tool to prevent the apoptosis in Sertoli cells.

**Figure 1:**
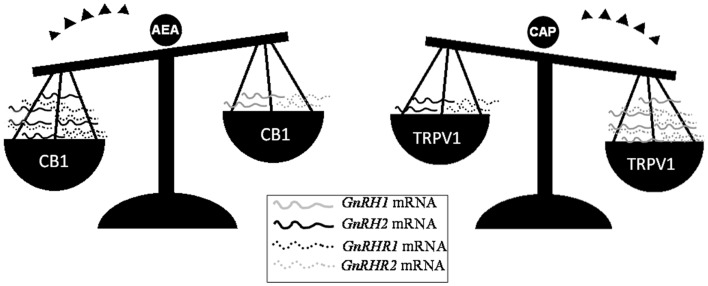
**Differential expression of GnRH system components after *in vitro* incubation of frog testis collected in post-reproductive periods with AEA or capsaicin (CAP), a selective agonist of TRPV1 receptor**. Since intracellular AEA also bind TRPV1, the involvement of AEA in the modulation of testicular GnRH signaling may occur via the selective activation of different eCB receptors.

Beside the activity exerted on Sertoli cells, eCBs are critical for the progression of spermatogenesis from mitotic stages throughout the meiotic stages and spermiogenesis events. In such a context, the FAAH-dependent modulation of eCB tone and the cell-specific expression of CB1, CB2, and TRVP1 provide evidence of multiple, differential eCB-dependent signaling involved in the spermatogenetic events. In mouse, decreasing levels of 2-AG have been detected from spermatogonia (SPG) to spermatocytes (SPC) and spermatids (SPT), suggesting that 2-AG, through CB2 – the receptor highly expressed just in mitotic and meiotic stages, but retained in residual body during the spermiogenesis – may act as an autocrine/paracrine mediator during spermatogenesis (73). Conversely, the high expression of *Trpv1* observed in meiotic stages (73) and the massive germ cell depletion detected in *Trpv1* null mice (96) candidate TRPV1 as a controller of meiotic stages. Very recently, the involvement of both CB1 and TRPV1 in the opposite modulation of testicular GnRH signaling (15, 68, 97) – a master system involved in the control of both spermatogenesis progression and steroidogenetic activity – has been reported in the anuran amphibian, the frog *R. esculenta* (97), a seasonal breeder in which two GnRH molecular forms (GnRH-I and GnRH-II) and three GnRH receptors (GnRH-RI, -RII and -RIII) have been characterized in testis (68). In such a context, AEA might act as an autocrine/paracrine factor via CB1 and as an intracrine signal via TRPV1; thus, it might be hypothesized that AEA, through the activation of specific receptors, switches on/off testicular GnRH signaling, leading to germ cell progression (Figure 1).

However, in mammalian and non-mammalian vertebrates, CB1 activity is linked to the control of post-meiotic stages (32, 65, 69, 73). In particular, it has been suggested that ECS controls different steps of spermiogenesis that is the phase of spermatogenesis consisting in the differentiation of SPT in SPZ. In particular, post-meiotic haploid round spermatids (rSPT) undergo biochemical and morphological changes becoming elongated cells (eSPT) and then SPZ. Sperm cells are differentially released from Sertoli cells by spermiation, a process characterized by species-specific features (65, 98). In mammals, SPZ undergo further transformations in the epididymis, which enables SPZ for fertilization (76, 77). These cellular modifications, and in particular some structural changes observed in SPT (i.e., acrosome development, nuclear shaping and chromatin condensation), seem to be related to ECS and in particular to CB1 activity.

A detailed immunolocalization of CB1 has been reported in rat SPT. CB1 appears in rSPT, around the nucleus, during acrosome development; the signal is retained in the head of elongating and condensing SPT, always close to the acrosome region, suggesting a role for CB1 in spermiogenesis, probably in chromatin packaging and in acrosome and/or cellular shape configuration (57, 72, 81). In agreement, several data demonstrate that CB1 regulates acrosome reaction, chromatin condensation, and nuclear size of SPZ (82, 99). Recent observations demonstrate that CB1 is involved in chromatin remodeling of SPT. In fact, during spermiogenesis, as the nucleus elongates and assumes a specie-specific shape, the chromatin condenses. It is worth noting that chromatin condensation differentially occurs, depending on the species. In mammals, chromatin condensation starts in eSPT producing condensing and then condensed SPT, which are mature elongated cells with strongly packaged chromatin (100). Many events characterize these chromatin cyto-architecture changes (101). Early during spermiogenesis, it is possible to observe the expression and storage of specific proteins involved in condensation and in DNA integrity maintenance, such as transition proteins (TNPs) and protamines (PRMs) (102). Others events concern the following: (i) displacement and degradation of the nucleosome structure; (ii) histone replacement by TNPs and then by PRMs; (iii) transcriptional silencing; (iv) DNA repair; and finally, (v) repackaging of the protaminated chromatin into toroidal structures (103, 104) [for recent review, see Ref. (94)]. These events strongly preserve DNA by damage and are involved in mechanism related to sperm maturation. Indeed, it is well known that inefficient expression or activity of TNPs/PRMs deranges histone displacement and causes production of SPZ with histone retention, incomplete chromatin condensation, and DNA damage (74, 105, 106). In both humans and rodents, abnormal levels of sperm DNA damage are associated with lower conception, implantation, and fecundity rates, and with higher miscarriage probability (95, 107, 108). In this context, Chioccarelli et al. (74) showed that *Cb1* gene deletion negatively influences chromatin remodeling in SPT, by reducing either transition protein 2 (Tnp2) levels or histone displacement. Secondary effects, related to the inefficient histone displacement (i.e., histone retention, uncondensed chromatin, DNA damage, and nuclear size elongation) have been postulated (22, 74). In agreement, *in vivo* and *in vitro* experiments show that AEA is able to act locally and upregulate *Tnp2* mRNA levels through CB1, via PKC/PKA pathways (17, 74). Furthermore, in *caput* epididymis from *CB1*^−/−^ mice, the percentage of SPZ retaining histones as well as the percentage of SPZ with uncondensed chromatin or with DNA damage, is higher as compared to normal mice. Interestingly, DNA damage increased during the epididymal transit, from *caput* to *cauda*, suggesting that CB1 preserve sperm DNA integrity of SPZ during epidydimal transit (74).

Recently, it has been demonstrated that estradiol, probably via stimulatory effects on FSH secretion and/or directly via paracrine actions within the testis, preserve chromatin condensation, and DNA integrity of SPZ, likely by promoting histone displacement in SPT (99). Indeed, it has been reported that *CB1*^−/−^ male mice show low levels of circulating E_2_, and when treated with 17β-estradiol, they rescue sperm chromatin quality by restoring histone content, chromatin packaging, DNA integrity, and nuclear length of SPZ (22, 99). These results corroborate the intriguing findings that the small nucleus of SPZ, containing chromatin that did not retain histones, appear fully condensed and able to preserve DNA from damage. On the contrary, the longer nucleus of SPZ, containing chromatin that retained histones, is uncondensed and unable to avoid DNA damage. The emerging exciting idea is that sperm nuclear dimensions can be a good marker for SPZ chromatin quality useful to select the SPZ qualitatively suitable for intracytoplasmic sperm injection (99).

## Conflict of Interest Statement

The authors declare that the research was conducted in the absence of any commercial or financial relationships that could be construed as a potential conflict of interest.
